# Prognostic factors after resection of locally advanced non-functional pancreatic neuroendocrine neoplasm: an analysis from the German Cancer Registry Group of the Society of German Tumor Centers

**DOI:** 10.1007/s00432-023-04785-0

**Published:** 2023-04-24

**Authors:** Thaer S. A. Abdalla, Monika Klinkhammer-Schalke, Sylke Ruth Zeissig, Kees Kleihues-van Tol, Kim C. Honselmann, Rüdiger Braun, Louisa Bolm, Hryhoriy Lapshyn, Stanislav Litkevych, Sergii Zemskov, Nehara Begum, Birte Kulemann, Richard Hummel, Ulrich Friedrich Wellner, Tobias Keck, Steffen Deichmann

**Affiliations:** 1 Department of Surgery, University Medical Center Schleswig-Holstein, Campus Lübeck, Ratzeburger Allee 160, 23564 Lübeck, Germany; 2 Network for Care, Quality and Research in Oncology (ADT), German Cancer Registry Group of the Society of German Tumor Centers, Berlin, Germany; 3 Institute of Clinical Epidemiology and Biometry (ICE-B), University of Würzburg, Würzburg, Germany; 4 Department of General Surgery, Bogomolets National Medical University, Kiev, 01601 Ukraine; 5 Department of Surgery, Johannes-Wesling-Klinikum Minden, Minden, Germany

**Keywords:** Pancreatic neuroendocrine neoplasms, Resection margin, Prognostic factors, Population-based analysis

## Abstract

**Objective:**

The available literature regarding outcome after pancreatic resection in locally advanced non-functional pNEN (LA-pNEN) is sparse. Therefore, this study evaluates the current survival outcomes and prognostic factors in after resection of LA-pNEN.

**Materials and methods:**

This population-based analysis was derived from 17 German cancer registries from 2000 to 2019. Patients with upfront resected non-functional non-metastatic LA-pNEN were included.

**Results:**

Out of 2776 patients with pNEN, 277 met the inclusion criteria. 137 (45%) of the patients were female. The median age was 63 ± 18 years. Lymph node metastasis was present in 45%. G1, G2 and G3 pNEN were found in 39%, 47% and 14% of the patients, respectively. Resection of LA-pNEN resulted in favorable 3-, 5- and 10-year overall survival of 79%, 74%, and 47%. Positive resection margin was the only potentially modifiable independent prognostic factor for overall survival (HR 1.93, 95% CI 1.71–3.69, *p* value = 0.046), whereas tumor grade G3 (HR 5.26, 95% CI 2.09–13.25, *p* value < 0.001) and lymphangiosis (HR 2.35, 95% CI 1.20–4.59, *p* value = 0.012) were the only independent prognostic factors for disease-free survival.

**Conclusion:**

Resection of LA-pNEN is feasible and associated with favorable overall survival. G1 LA-pNEN with negative resection margins and absence of lymph node metastasis and lymphangiosis might be considered as cured, while those not fulfilling these criteria might be considered as a high-risk group for disease progression. Herein, negative resection margins represent the only potentially modifiable prognostic factor in LA-pNEN but seem to be influenced by tumor grade.

**Supplementary Information:**

The online version contains supplementary material available at 10.1007/s00432-023-04785-0.

## Introduction

Pancreatic neuroendocrine neoplasms (pNEN) are rare pancreatic tumors showing increased incidence over the past few decades. pNEN represent about 7% of all pancreatic tumors (Yadav et al. 2018). According to the European Neuroendocrine Tumor Society (ENETS) guidelines, there are two main forms of pNENs: functional pNEN and non-functional (NF) pNEN (Partelli et al. 2017). Unlike functional pNEN, which are hormonally active and are, therefore, often detected early in the course of the disease, NF-pNENs often remain asymptomatic if not incidentally discovered, reaching significant tumor burden until causing symptoms related to the mass effect of the tumor. Therefore, 65% of NF-pNENs are locally advanced or metastatic at presentation (Cloyd and Poultsides 2015).

Despite remarkable advances in the field of oncology and the introduction of novel multimodal treatment protocols, surgical resection remains the only curative treatment of pNEN (Howe et al. 2020; Pavel et al. 2020). The low incidence of pNEN, the wide heterogeneity regarding tumor grade and stage as well as the variety of treatments available today are the main reasons behind the lack of evidence on various aspects of surgical treatment (Jensen et al. 2019).

Until now, the definition of locally advanced pNEN is still controversial and not unified. Most studies included large pNEN with locoregional involvement or oligometastatic disease (Norton et al. 2003; Squires et al. 2020; Fusai et al. 2021). It was first Titan et al. (2020) who defined LA-pNEN as tumors larger than 4 cm (T3/T4) without the presence of distant metastasis. In 2018, the ENETS reported the main areas of unmet needs in the management of patients with functional and non-functional pNEN. Assessment of surgery and identification of prognostic factors in resectable non-metastatic locally advanced pNEN was highlighted as an important aspect requiring further clarification (Jensen et al. 2019).

Due to the low incidence of pNEN in general and this clinical category in specific, multicentric and population-based registries represent an invaluable source of information. This study aimed to evaluate the clinical outcome and prognostic factors in patients after resection of locally advanced non-metastatic non-functional pancreatic neoplasms (LA-pNEN) using data from population-based cancer registries in Germany.

## Materials and methods

### Patient cohort and study design

This is a retrospective analysis of pooled anonymized cancer registry data. The data were derived from 17 population-based clinical cancer registries participating in the German Cancer Registry Group of the Society of German Tumor Centers (ADT), covering the time period from 2000 to 2019. The registry data were used according to the regulations of the ADT. The study was approved by the ethics committee of the University of Lübeck, Germany #20-319.

Of all patients with pancreatic malignancy (codes C25.0–C25.9, ICD-O 3. Edition (ICD-O-3)) patients with non-functional pNEN (ICD-O-3 morphology code 8240-1/3, 8246 and 8249/3) were extracted. Patients with any type of functional pNEN (8150-3, 8151-3, 8152-3, 8153-3, 8154-3, 8155-3, and 8156-3) such as insulinomas, glucagonomas, islet cell carcinoma, VIPoma, somatostatinoma, mixed neuroendocrine non-neuroendocrine neoplasia, and Gastrinomas were excluded (World Health 2013). Further, only patients with LA-pNEN, defined as T stage T3 or T4 with or without lymph node metastasis and without distant metastasis (M0), were included. Only patients who underwent upfront surgery (*n* = 277) were included in the survival analysis.

The following parameters were retrieved from the cancer registry data: sex, age at diagnosis (years), lymph node metastases (N0, N+), T stage (T3–T4), lymphangiosis (L0, L1), hemangiosis (V0, V1), tumor grade (G1–G3), resection margin status (R0, R1, R2), tumor location (pancreatic head, body, and tail), type of therapy (best supportive care, operation alone, neoadjuvant chemotherapy/radiochemotherapy plus operation with/without adjuvant therapy, operation plus adjuvant chemotherapy/radiochemotherapy, chemotherapy/radiochemotherapy alone), operation type (pancreatoduodenectomy, distal pancreatectomy, total pancreatectomy), follow-up time (months after diagnosis), and status at last follow-up (dead/alive and disease recurrence status).

The variables age, lymph node metastasis, tumor location, and resection status were dichotomized as follows: age ≤ 65 years versus > 65 years, lymph node metastasis N0 vs N+ , tumor location (tail vs head and body) and resection status negative (R0) versus positive (R1/R2). The pNEN TNM classification underwent changes over the selected study period (2000–2019). The main change was regarding T stage in T3 and T4 tumors, where the definition differed according to extra-pancreatic organ involvement. As metric tumor size and the exact tumor extension was not available in our dataset, restaging according to the current TNM classification was only possible by combining T stages T3 and T4 into one group, therefore, including all patients regardless of organ involvement and time period (Sobin et al. 2011; Brierley et al. 2017; Cong et al. 2018).

### Statistical methods

For statistical analysis, SPSS 26 for Windows (Armonk, NY, USA) was used. For descriptive statistics, absolute and relative frequencies, median and interquartile range as well as Kaplan–Meier estimates and plots were used. Overall survival was calculated as the time period from the date of diagnosis to the date of death. Disease-free survival was defined as the period from the date of tumor resection to the date of local or metastatic recurrence. Statistical testing was performed by Chi squared test, Logrank test and Cox regression using a two-sided significance level of *p* = 0.05.

## Results

### Patient cohort

From 2000 to 2019, *N* = 2446 patients with diagnosis of pNEN were identified. Of those, 280 patients met the inclusion criteria for LA-pNEN. In this cohort, the median age was 63 ± 18 (median ± IQR). There were 155 (56%) patients under 65 years and 122 (44%) females. Data regarding tumor location were available in 207 patients. The tumor was located in the pancreatic head in 106 (51%) patients, in the pancreatic body in 33 (16%) patients, and in the pancreatic tail in 70 (34%) patients. The mean follow-up time was 43 months (95% CI 37–48 months).

### Treatment and histopathology

*N* = 277 patients underwent upfront surgery. Three patients underwent neoadjuvant therapy followed by surgical resection. The type of resection was reported in 183 patients: 91 patients underwent pancreatic head resection, 82 patients underwent distal pancreatectomy, and 10 had a total pancreatectomy.

Lymph node metastasis was present in 122 (45%) of the patients. G1 pNEN was present in 39% of the patients, G2 pNEN in 47%, and G3 pNEN in 14% (Table 1). Moreover, advanced tumor grade was associated with patients older than 65 years (*p* = 0.020) and with tumors located in pancreatic head (*p* = 0.005) (Table S1).

**Table 1:** Patient cohort parameters

Variables	*n* (%), Median ± IQR
Sex	
Female	125 (45%)
Male	152 (55%)
Age	63 ± 18 years
Grade of differentiation	
G1	100 (39%)
G2	121 (47%)
G3	36 (14%)
Lymph node metastasis	
Negative	151 (55%)
Positive	122 (45%)
Margin (R) status	
R0	185 (89%)
R1	21 (10%)
R2	2 (1%)
Lymphangiosis (L)	
L0	121 (55%)
L1	97 (45%)
Hemangiosis (V)	
V0	151 (70%)
V1	64 (30%)
Perineural Invasion (Pn)	
Pn0	71 (59%)
Pn1	48 (41%)
Tumor location within the pancreas	
Head	106 (51%)
Body	33 (16%)
Tail	68 (33%)

*n*, patient number, *()* percentage, *IQR* interquartile range, *R-status* resection margin status

R0 resection was achieved in 185 (89%), R1 resection in 21 (10%) and R2 resection in two patients. Positive margin status was not associated with tumor location (*p* = 0.751) but was associated with higher tumor grade (*p* = 0.020) (Table S1).

### Overall survival

**Fig. 1:**
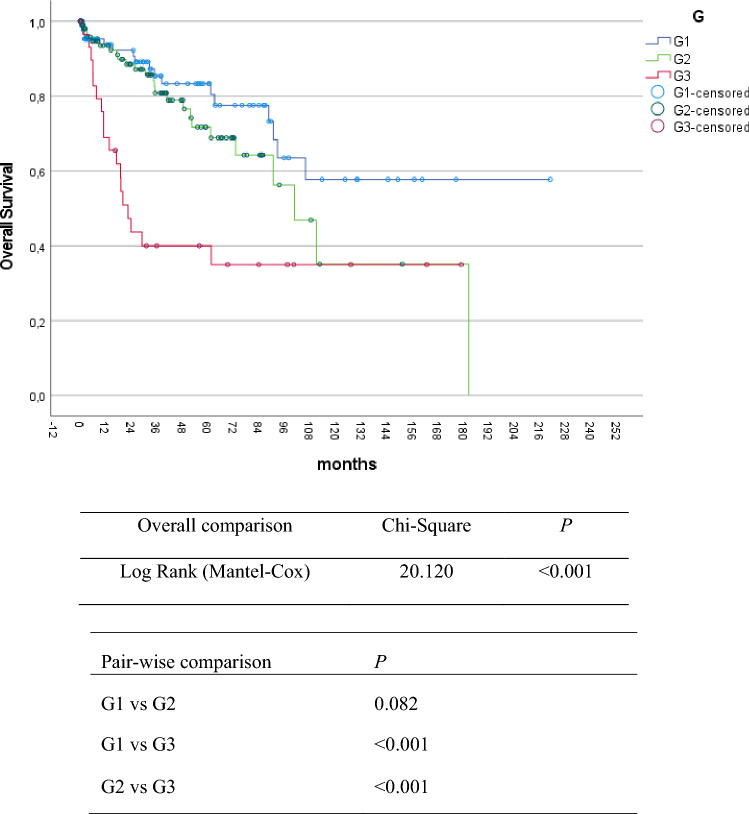
Kaplan–Meier plot for overall survival according to tumor differentiation in locally advanced pNEN

**Fig. 2:**
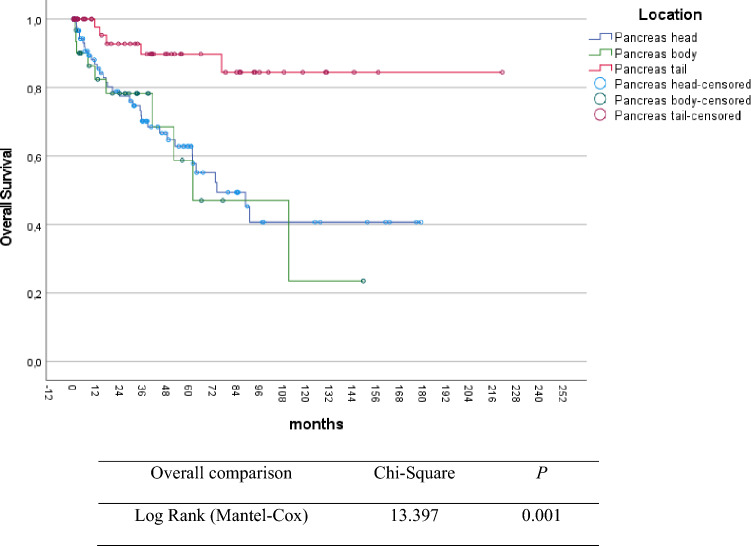
Kaplan–Meier plot for overall survival according to tumor location in locally advanced pNEN

**Fig. 3:**
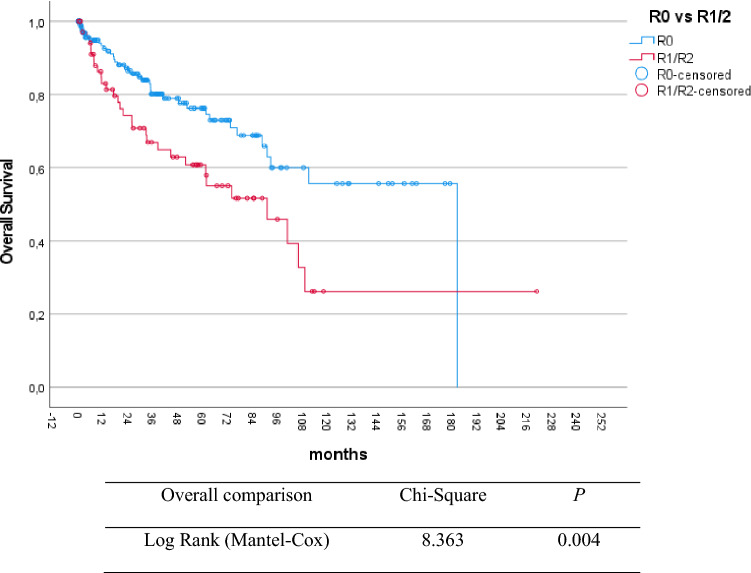
Kaplan–Meier plot for overall survival according to resection margin in locally advanced pNEN

277 patients with upfront resection were included in the survival analysis. The 3-year, 5-year, and 10-year overall survival in resected LA-pNEN were 79%, 74%, and 47%, respectively. Univariable analysis demonstrated that age below 65 years (*p* = 0.004), low tumor grade (G1) (*p* = 0.007), margin free resection (R0) (*p* = 0.004) and tumor location in the pancreatic tail (*p* = 0.002) were associated with longer overall survival (Figs. 1, 2, 3, and Table 2).

**Table 2:** Overall survival according to the different histopathological factors

Variables	3-year	5-year	10-year	Median OS (months)	*p*
Grade of differentiation					< 0.001
G1	86%	83%	59%	204	
G2	81%	72%	36%	104	
G3	40%	40%	35%	22	
Lymph node metastasis					0.713
Negative	79%	78%	42%	101	
Positive	74%	67%	48%	107	
Resection margin status					0.009
R0	80%	77%	56%	181	
R1/R2	67%	61%	33%	83	

Association of overall survival depending on different histopathological variables. Median OS in months. *p* value derived from Log Rank test for overall survival

In multivariable analysis, advanced age (HR 2.28, 95% CI 1.19–4.37, *p* value = 0.012), tumor location in the pancreatic head or body (HR 5.65, 95% CI 1.71–18.58, *p* value = 0.004) and positive resection margins (HR 1.93, 95% CI 1.71–3.69, *p* value = 0.046) were independent negative prognostic factors in patients after resection of LA-pNEN (Table 3). In a subgroup analysis for patients with follow-up longer than 90 days, age over 65 years (HR 2.48 95% CI 1.26–4.88), positive resection margin (HR 2.44, 95% CI 1.24–4.81), tumors located in pancreatic body and head (HR 4.56, 95% CI 1.37–15.13) as well as and G3 tumors (HR 2.73, 95% CI 1.12–6.66) resulted to be independent negative prognostic factors for overall survival (Table S3).

**Table 3:** Univariable and multivariable analyses for overall survival in upfront resected locally advanced pNEN

	Univariable analysis				Multivariable analysis (*n* = 162)		
	*n*	HR	95% CI	*p*	HR	95% CI	*p*
Age, ≥ 65 vs < 65	277	2.10	1.29–3.42	0.003	2.28	1.19–4.37	0.012
Sex, male vs female	277	0.84	0.52–1.37	0.502	0.84	0.44–1.62	0.613
Tumor grade (G)	257			< 0.001			0.281
G2 vs G1		1.44	0.77–2.68	0.244	1.21	0.57–2.60	0.609
G3 vs G1		3.77	1.93–7.35	< 0.001	1.93	0.82–4.50	0.128
Lymph node metastasis	273	1.09	0.67–1.76	0.713			
Margin status, R1/R2 vs R0	208	1.93	1.20–3.10	0.006	1.93	1.01–3.69	0.046
Lymphangiosis (L0 vs L1)	218	0.94	0.55–1.74	0.949			
Hemangiosis (V0 vs V1)	215	0.70	0.37–1.33	0.285			
Tumor location, body/head vs tail	207	4.65	1.8–11.74	0.001	5.65	1.71–18.58	0.004

*p *value derived from Cox regression analysis

*HR* hazard ratio

### Disease-free survival analysis

Information regarding disease-free survival was available in 209 patients. The 3-year, 5-year, and 10-year disease-free survival in patients after upfront resection of LA-pNEN was 54%, 41%, and 22%, respectively. Univariable analysis disclosed that lymphangiosis (*p* = 0.002), advanced tumor grade (*p* = 0.003) and the presence of lymph node metastasis (*p* = 0.020) were associated with shorter disease-free survival (Table 4 and Figs. S1–S3).

**Table 4:** Univariable and multivariable analyses for disease-free survival after upfront resection of locally advanced pNEN

	Univariable analysis				Multivariable analysis (*n* = 105)		
	*n*	HR	95% CI	*p*	HR	95% CI	*p*
Age, < 65 vs ≥ 65	151	0.98	0.60–1.59	0.934	0.83	0.45–1.54	0.545
Sex, male vs female	151	1.07	0.66–1.71	0.777	1.24	0.71–2.17	0.442
Tumor grade G2/3 vs G1	130	1.68	1.01–2.79	0.043	1.72	0.89–3.37	0.107
Nodal involvement	150	1.76	1.0–2.86	0.022	1.41	0.77–2.57	0.256
Resection status R1/R2 vs R0	150	1.35	0.72–2.52	0.342			
Lymphangiosis (L1 vs L0)	112	2.43	1.35–4.38	0.003	2.10	1.11–3.98	0.023
Tumor location, body/head vs tail	106	1.02	0.59–1.76	0.932			

*p *value derived from Cox regression analysis

*HR* hazard ratio

Multivariable analysis (*n* = 105) demonstrated advanced tumor grade G3 (HR 5.26, 95% CI 2.09–13.25, *p* value < 0.001) and lymphangiosis (HR 2.35, 95% CI 1.20–4.59, *p* value = 0.012) to be the only independent negative prognostic factors for disease-free survival in patients after upfront resection in LA-pNEN (Table 4).

### Recurrence in patients with positive prognostic factors

In the subgroup of *n* = 71 patients with negative resection margins (R0) and absence of lymph node metastasis and lymphangiosis (N0, L0), only one patient developed local recurrence and 10 patients developed distant metastasis during follow-up (Table S2). The incidence of distant metastasis increased with advanced tumor grade (3% of G1, 25% of G2 and 60% of G3 tumors).

## Discussion

The results of this study identified major aspects regarding survival in patients after resection of non-metastatic locally advanced pNEN. First, this study demonstrates that patients with LA-pNEN have favorable long-term 3-, 5-, and 10-year overall survival of 79%, 74%, and 47% respectively, which highlights the importance of offering curative intent surgical therapy at the time of diagnosis.

Furthermore, our study demonstrated that positive resection margins (R1/2), tumor location in the pancreatic head or body, and advanced age (> 65 years) were the only independent negative prognostic factors for overall survival in this cohort. Herein, resection margin status represents the only potentially modifiable factor for overall survival in LA-pNEN (Tables 3 and S4). Negative margin status was associated with 5- and 10-year overall survival of 77% and 56%, compared to 61% and 23% in patients with R1/R2 status (Table 2). For this reason, patients with LA-pNEN should be treated in high-volume centers with experience in multivisceral resections and vascular reconstruction to maximize the possibility of R0 resection.

In the current study, 45% of the patients had positive lymph node metastasis. Unlike small pNEN (< 2 cm) were the incidence of LNM in G1 pNEN is negligible compared to G2–G3 tumors (3% vs 16–100%) (Sallinen et al. 2018). In our study, lymph node metastasis was present in 37% of G1, 46% of G2 and 57% of G3 of LA-PNEN (Table S1). Interestingly, lymph node metastasis was not associated with overall survival. However, lymph node metastasis and lymphangiosis, as well as advanced tumor grade, were associated with shorter disease-free survival. Furthermore, we identified G3 pNEN and lymphangiosis as the only independent negative prognostic factors of disease-free survival in LA-pNEN. Since higher tumor grade was also associated with positive resection margin, we can suggest that the grade of differentiation relates to lymphatic tumor spread and locoregional tumor dissemination, therefore, leads to positive resection margin and recurrence. This implies also that lymphadenectomy should be carried out in LA-pNEN to achieve local control and avoid tumor recurrence after curative resection. Another observation is that, after lymphadenectomy and resection of positive lymph nodes, recurrence and eventually death occurs relatively late due to the slow-growing nature of pNEN. Therefore, long follow-up times are needed to analyze overall survival, but tumor biology is probably better reflected in disease-free survival at earlier time points. This might explain the observed differences in prognostic factors of OS and DFS.

In the subgroup analysis of patients with favorable prognostic factors (R0, N0, L0, *n* = 71), only one patient developed local recurrence and 10 patients developed distant metastasis during follow-up (Table S2). Almost all recurrences occurred in G2 and G3 tumors. This means, cure can be achieved in G1 LA-pNEN in the presence of favorable histopathological factors such as L0, N0, and margin negative resection (R0). As such, resection margin, grade of differentiation, lymph node metastasis and lymphangiosis may also serve as prognostic factors to identify patients with a high-risk for metastasis and local recurrence.

LA-pNENs represent a subgroup of patients with a high risk for vascular involvement and multivisceral resection during exploration. Fusai et al. reported that 25% of T3–T4 pNEN required portal vein reconstruction, compared to only 3% in patients with T1–T2 pNEN in the same cohort (Fusai et al. 2021). Similarly, Titan et al. reported that 25% of LA-pNENs were associated with vascular invasion in the radiological studies, and 17% required eventually vascular reconstruction (Titan et al. 2020). They also reported a very high 10-year OS of 91% compared to our results of 47%. This could be due to the differences in multimodal therapy regimes, differences in the age groups (57 vs 63 years), and tumor grading (G1 54% vs 39%, G2 30% vs 47%, and G3 1% vs 14%), and more importantly, because they included functional pNEN such as insulinomas (*n* = 9) in their cohort, which are known to have excellent prognosis after curative resection (Roland et al. 2012; Kasumova et al. 2017). Unfortunately, the details of surgical resection (i.e., extent of multivisceral resection, extent of lymphadenectomy) and reconstruction are not available in the cancer registry data used in this study. However, due to comparable R0 resections, we can assume that similar surgical approaches (89% vs 84%) have been adopted in the treatment of LA-pNEN.

Another possibility is to offer patients with LA-pNEN neoadjuvant treatment, analogous to patients with borderline resectable pancreatic ductal adenocarcinoma (BR-PDAC). Although this treatment strategy is not well established in pNEN, several agents have demonstrated promising results, including systemic chemotherapies (Prakash et al. 2017; Squires et al. 2020), PRRT (Parghane et al. 2021; Zanata et al. 2021), and targeted therapy such as everolimus (Sato et al. 2017). These protocols could offer a solution to increase resectability even in initially unresectable pNENs.

Several limitations should be considered when interpreting our results. Although the registry data are the result of multi-institutional collaboration, possible inconsistency in patient selection, surgical experience, pathologic assessment, and reporting likely existed. Moreover, the German Clinical Cancer Registry Group collects data from several regional clinical cancer registries implicating data entry by different people in variable quality. Furthermore, the registry data did not include detailed information regarding treatment schemes, timing, and completion of these protocols in pNENs or surgery-related complications, such as pancreatic fistula or postoperative morbidity. In addition, information regarding Ki67% or the differentiation between pNEN G3 or NEC was not available in queried dataset. Nevertheless, this is the largest and the first registry-based analysis of treatment and survival outcomes of locally advanced non-functional pNEN without distant metastasis. Moreover, we demonstrated the favorable prognosis of LA-pNEN and identified the involvement of resection margins as the only modifiable prognosticator in this subgroup of patients. Future studies might address the impact of neoadjuvant therapy on resection status and the relevance of circumferential resection margin on prognosis in LA-pNEN.

## Conclusion

Resection of locally advanced pNEN with negative resection margins is associated with favorable 5- and 10-year overall survival (79% and 47%). The resection margin was the only potentially modifiable prognostic factor for LA-pNEN. Positive lymph node metastasis was present in almost half of the patients; therefore, resection with curative intent and radical lymphadenectomy should be routinely performed in LA-pNEN. Patients after resection of LA-pNEN with R0 N0 L0 G1 histopathology might be considered cured, while patients not meeting these criteria to be categorized as high-risk patients for local recurrence and distant metastasis.

## Supplementary Information

Below is the link to the electronic supplementary material.

Supplementary file1 (DOCX 79 KB)

## Data Availability

Data were obtained from German Cancer Registry Group of the Society of German Tumor Centers (ADT) and are available upon request from and under the regulations of the Society of German Tumor Centers.
