# Supplementary material for: Prognostic factors after resection of locally advanced non-functional pancreatic neuroendocrine neoplasm: an analysis from the German Cancer Registry Group of the Society of German Tumor Centers
Source: J Cancer Res Clin Oncol. 2023 Apr 24;149(11):8535–43. doi: 10.1007/s00432-023-04785-0 (PMC10374814; doi:10.1007/s00432-023-04785-0)
Supplement: Supplementary file 1 — Supplementary file1 (DOCX 79 KB) [file 432_2023_4785_MOESM1_ESM.docx]

**Supplementary Information:**

**Journal of Cancer Research and Clinical Oncology**

**Title: Prognostic factors after resection of locally advanced non-functional pancreatic neuroendocrine neoplasm – an analysis from the German Cancer Registry Group of the Society of German Tumor Centers**

Thaer S. A. Abdalla^1^, Monika Klinkhammer-Schalke^2^, Sylke Ruth Zeissig^2,3^, Kees Kleihues-van Tol^2^, Kim C. Honselmann^1^, Rüdiger Braun^1^, Louisa Bolm^1^, Hryhoriy Lapshyn^1^, Stanislav Litkevych^1^, Sergii Zemskov^4^, Nehara Begum^1,5^, Birte Kulemann^1^, Richard Hummel^1^, Ulrich Friedrich Wellner^1^, Tobias Keck^1^, Steffen Deichmann^1^

^1^ Department of Surgery, University Medical Center Schleswig-Holstein, Campus Lübeck, Lübeck, Germany

^2^ Network for Care, Quality and Research in Oncology (ADT), German Cancer Registry Group of the Society of German Tumor Centers, Berlin, Germany

^3^ Institute of Clinical Epidemiology and Biometry (ICE-B), University of Würzburg, Würzburg, Germany

^4^ Department of General Surgery, Bogomolets National Medical University, 01601 Kiev, Ukraine

^5^ Department of Surgery, Johannes-Wesling-Klinikum Minden, Minden, Germany

Correspondence during submission:

Thaer S. A. Abdalla

Department of Surgery

University Medical Center Schleswig Holstein, Campus Lübeck

Ratzeburger Allee 160

23564 Lübeck, Germany

Email: [thaer.abdalla@uksh.de](about:blank)

ORCID: 0000-0002-0448-2225

Table S1: Association of tumor grading to different parameters in LA-pNEN (n=277)

| Variable | G1 | G2 | G3 | p-value |
| --- | --- | --- | --- | --- |
| Age  <65  >65 | 66 (65%)  36 (35%) | 60 (49%)  63 (51%) | 16 (43%)  21 (57%) | 0.020 |
| Lymph node metastasis  negative  positive | 61 (63%)  36 (37%) | 66 (55%)  55 (46%) | 16 (43%)  21 (57%) | 0.110 |
| Tumor location  Head  Body  Tail | 35 (49%)  11 (16%)  25 (35%) | 42 (42%)  21 (21%)  36 (36%) | 23 (82%)  3 (11%)  2 (7%) | 0.005 |
| Resection margin  positive  negative | 25 (25%)  77 (76%) | 44 (36%)  79 (64%) | 18 (49%)  19 (51%) | 0.020 |

Legend: *P*-value derived from χ2 test

Table S2: Incidence of local recurrence and metastasis in patients with **LA-PNEN with N0, L0 and R0** status according to the grade of differentiation.

| Variables | G1 | G2 | G3 |
| --- | --- | --- | --- |
| Local recurrence | 0/34 | 1/28 | 0/5 |
| Distant metastasis | 1/31 | 6/24 | 3/5 |

Table S3: Multivariable analysis for overall survival after upfront resection of locally advanced pNEN in patients with a follow-up longer than 90 days (n=143).

|  | Multivariable Analysis | | |
| --- | --- | --- | --- |
| Variables | HR | 95% Cl | *p* |
| Age, <65 vs ≥65 | 2.48 | 1.26-4.88 | 0.008 |
| Sex, male vs female | 1.07 | 0.55-2.07 | 0.840 |
| Grade of Differentiation, G |  |  | 0.068 |
| G2 vs G1 | 1.39 | 0.61-3.184 | 0.425 |
| G3 vs G1 | 2.73 | 1.12-6.66 | 0.027 |
| Margin status, R1/R2 vs R0 | 2.44 | 1.24-4.81 | 0.009 |
| Tumor location, Body/Head vs Tail | 4.56 | 1.37-15.13 | 0.013 |

# Legend: *p-value derived from* Cox regression analysis, HR hazard ratio

Figure S1: Kaplan-Meier plot for disease-free survival according to grade of differentiation in locally advanced pNEN


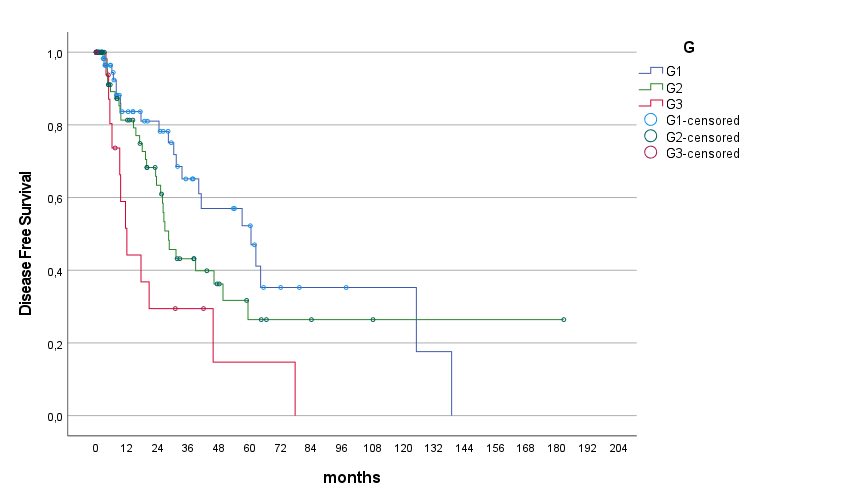


| Overall comparison | Chi-Square | *P* |
| --- | --- | --- |
| Log Rank (Mantel-Cox) | 11.676 | 0.003 |

| Pair-wise comparison | *P* |
| --- | --- |
| G1 vs G2 | 0.116 |
| G1 vs G3 | <0.001 |
| G2 vs G3 | 0.028 |

Figure S2: Kaplan-Meier plot for disease-free survival according to the presence of lymphangiosis in locally advanced pNEN


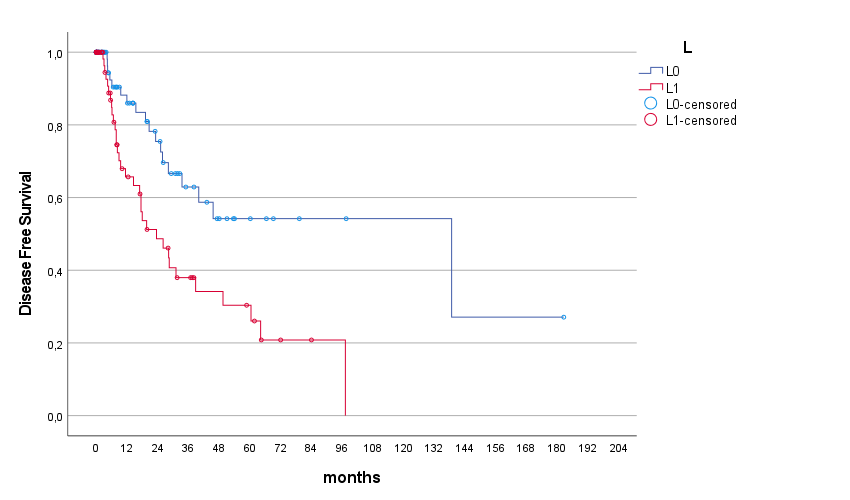


| Overall comparison | Chi-Square | *P* |
| --- | --- | --- |
| Log Rank (Mantel-Cox) | 8.429 | 0.004 |

Figure S3: Kaplan-Meier plot for disease-free survival according to the presence of lymph node metastasis in locally advanced pNEN


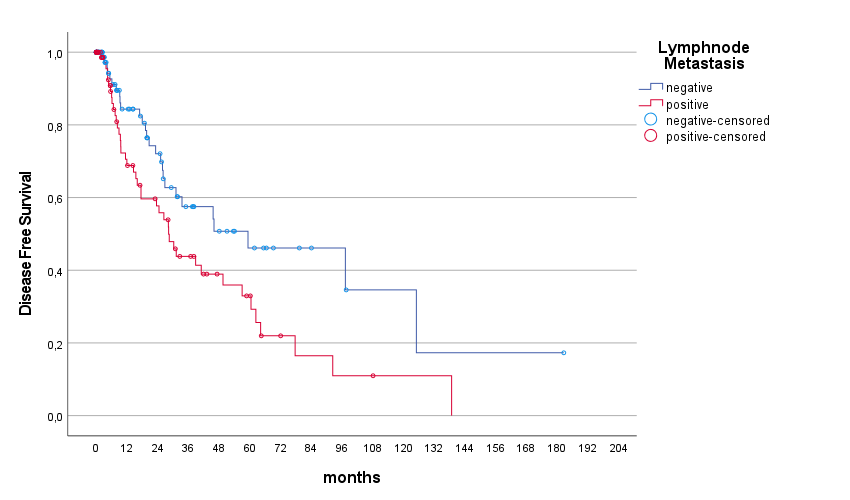


| Overall comparison | Chi-Square | *P* |
| --- | --- | --- |
| Log Rank (Mantel-Cox) | 4.900 | 0.027 |
